# Diabetic Ketoacidosis: An Emergency Medicine Simulation Scenario

**DOI:** 10.7759/cureus.1286

**Published:** 2017-05-29

**Authors:** Reuben Addison, Tate Skinner, Felix Zhou, Michael Parsons

**Affiliations:** 1 Human Kinetics and Recreation, Memorial University of Newfoundland; 2 Biochemistry Nutrition, Memorial University of Newfoundland; 3 Medical Education, Memorial University of Newfoundland; 4 Emergency Medicine, Memorial University of Newfoundland

**Keywords:** diabetic ketoacidosis, simulation, expertise, emergency medicine

## Abstract

Simulation provides a safe environment where learning is enhanced through the deliberate practice of skills and controlled management of a variety of clinical encounters. This is particularly important for core cases and low-frequency, high-stakes procedures and encounters. Competency-based medical education has seen widespread adoption in the field along with ongoing work in the areas of undergraduate and postgraduate training. Similarly, effective professional development activities stand to benefit greatly from a more stringent integration of simulation and competency-based approaches. This particularly makes sense when considering the goals of patient safety and achievement of optimal clinical outcomes. The current report describes a simulation training session designed to acquaint emergency medicine residents with the presentation and management of diabetic ketoacidosis (DKA) through the use of simulation.

## Introduction

Diabetic ketoacidosis (DKA) is a life-threatening complication of diabetes mellitus, most commonly occurring in patients with type I diabetes. As individuals with uncontrolled type I diabetes develop an insulin deficiency, the cells in their body are unable to utilize glucose properly to meet their energy demands. In response, the body begins to metabolize other nutrients (fat, proteins, glycogen) to prevent starvation. As a result of these metabolic pathways, acidic ketones are produced leading to metabolic acidosis that is characteristic of DKA. Some typical presenting symptoms include dehydration and altered mental status [1-3].

Roughly 30% of children with type 1 diabetes present with DKA at the time of their diagnosis [4]. DKA is the most common cause of hospitalization and mortality in children with type I diabetes, occurring in 1% to 10% of patients per year [5-6]. Initial presentation at a later age is less common and presenting symptoms can be confused with a variety of other conditions. Given the frequency with which diabetic patients present with DKA and the severe consequences if left untreated, it is vital that emergency department (ED) physicians are familiar with the presentation and management of this condition. The reader should keep in mind that the management of DKA in the pediatric population requires particular caution due to the risk of cerebral edema. This technical report describes a simulation teaching session on DKA that is designed for a cohort of post-graduate emergency medicine trainees. The objective of this case is to provide learners with the following knowledge/skills:

Objective 1: Assess the patient with an undifferentiated altered level of consciousness (LOC) - relevant to the CanMEDS Medical Expert role.

Objective 2: Diagnose and treat DKA - relevant to the CanMEDS Medical Expert role.

Objective 3: Demonstrate effective communication skills for team interactions and conflict management - relevant to the CanMEDS communicator, collaborator, and professional roles.

Simulation has developed as an important tool to complement traditional teaching methods. The traditional “see one, do one, teach one” approach has fallen out of favor. A transition to a more rigorous competency-based education and assessment system is well under way, with an increased focus on patient safety and quality assurance as they are important drivers of this change. Grant, et al. [7] provide a succinct review of the evolution and advancement of simulation to its current role and highlight its importance in medical education. They cite evidence to support the use of the safe and controlled environment of simulation. They also highlight its benefits in learning different skills from procedural tasks to more team-based and interpersonal skills. Simulation integrated with traditional teaching methods serves to supplement and enhance learning. In our program, we broadly work from the Kolb’s learning cycle framework [8]. We strive to create a “safe” learning environment where learners are active participants [9]. We do our best to optimize fidelity of the cases and enhance the emotional engagement of learners – aiming for a balance of “activation” and a “pleasant” experience as depicted in the circumplex model of affect [10-11]. 

## Technical report

### Case

A 19-year-old male presented to the ED with decreased LOC. The patient was found in his dorm room in the morning. An ambulance was called and the patient was transported to the hospital. Few historical details are available as the patient is from another province. Aside from that, he may have consumed some alcohol the previous night. The patient did attest he has no known past medical history. His family is not present with him and the emergency medical services (EMS) crew has departed. The patient is drowsy, but responsive (depending on the level of case difficulty chosen) and has some dried vomit on his clothing. The patient has just arrived in the ED and no interventions have yet been performed. There is a single nurse confederate to help with the case.

The Context, Input, Process, Product (CIPP) model is applied below to give a clear overview of the current case design and to demonstrate how to modify the case to meet the needs of various learners [12].

### Context

The case details can be adjusted to accommodate the available physical space, learners, modality, and the use of confederates. The case can be performed in a variety of settings including in-situ in the ED, clinic, or hospital ward. It can also be conducted in a simulation lab setting. The "difficulty" level can be easily modified to meet the needs of a variety of learners by adjusting case inputs, as outlined below in Table 1.

**Table 1:** Signal/noise modifiers for adjusting case complexity according to the level of the learner

Modality/Patient	High Fidelity	Standarized Patient (SP)	Standardized Patient + Task Trainer
SP confederates			
Nurse	+	+	+
Family	+/-	+/-	+/-
Case difficulty	Low	Moderate	Advanced
Setting/location	Tertiary referral.	Community hospital/limited backup.	Community hospital - little to no help available.
Information at initial presentation	Triage chart. Monitors + intravenous (IV) in place.	Limited triage chart. Monitor off.	No initial information. Patient unable to provide information. Must find identification and contacts in wallet & proceed to contact the family.
Patient modifiers			
Patient condition	Fairly well	Moderate	Sick
Patient Info			
History (Hx)	Patient can give Hx, including family contacts.	Limited. Need to use additional resources - call family/friends.	Only from outside contact to family. Check wallet, MedicAlert bracelet, etc.
Physical	Vitals borderline abnormal.	Tachy, drowsy.	Decreased level of consciousness, vitals abnormal.
Investigations			
Electrocardiography (ECG)	Sinus tachy	Sinus tachy, signs hyperK.	Sinus tachy + signs hyperK, other complex rhythms.
Point of Care Ultrasound (PoCUS)	Equivocal	Mild volume depletion.	Marked volume depletion.
Chest X-Ray (CXR)	Clear + or negative	Slight abnormality (e.g., pneumonia).	Equivocal
Procedures			
Intubation	N/A	N/A	Easy to challenging
Central line setup	N/A	+/- used	+/- used/difficult placement.

The advantages of a high-fidelity simulator can also be leveraged by utilizing standardized patients (SP) to fill the role of patients, nurses, and confederates to improve realism. A hybrid setup is sometimes applied during the scenario to include the challenge of performing procedures such as central line placement.

### Inputs

See Table 2 below.

**Table 2:** Inputs for managing diabetic ketoacidosis

Case setting	
A “limited-resource setting” or community hospital where the resident is the team lead and must handle the case as best as they can before calling for help. The physical room setup contains a high-fidelity mannequin with related systems and monitors. Alternatively, lower-tech options such as tablets and a computer screen may be used to display relevant vitals, documents, images and videos if resources are limited.	
Personnel	
- Simulation lab technologist (if a high-fidelity mannequin is used). - Confederates: Standardized patient (SP) nurse, SP family member, paramedic. - Facilitators: staff/faculty.	
Moulage	
- Dried vomit on patient’s shirt. Decreased level of consciousness.	
Supplies	
*The basics*	
- Blood pressure cuff, stethoscope, thermometer, sat probe, glucose monitor.	
*Procedural specifics (optional)*	
- High-fidelity (depending on procedures that can be done on the mannequin), central line task trainer, low-fidelity airway setup (for intubation).	
*Carts*	
i) Advanced Cardiovascular Life Support (ACLS) cart: defibrillator, medications, appropriate equipment	ii) Airway cart: a) Basic airway: Oxygen mask/tubing, bag valve mask (BVM), nasopharyngeal and oropharyngeal airways.
	b) Intubation: Endotracheal tubes & laryngoscopes (sized appropriately for the case), stylet, bougie, 10mL syringe, yankauer suction.
*Medications and fluids*	
- Blank medication bag labels. - 100 cc intravenous (IV) bags to use for “general” medication infusions. - Various IV bags (1000cc, 500cc normal saline). - Drugs (antibiotics, vasopressors).	
Investigations (refer to Table 1 for case variants)	
- Electrocardiography (ECG) - X-ray - Labs - Point of Care Ultrasound (PoCUS) images (normal if asks)	

### Process

A pre-briefing session is conducted prior to the start of the simulation scenario. The main goal is to establish a safe learning environment for the learner [9, 13]. The “fiction contract” is reviewed, addressing the limitations and ‘believability’ of the simulation scenario. The “basic assumption” – that everyone is present for the purpose of learning and are viewed as intelligent, capable individuals who will try their best  – is reviewed. The evaluative nature of the session, if any, is made clear from the beginning. Learners are provided with an introductory simulation lecture and an opportunity to become familiar with the simulation lab and equipment prior to the session, usually at the beginning of the academic year.

This case was designed by the ED faculty and follows a standard format provided by a simulation lab. Most cases are based on true ED patient encounters and scenarios are written along a spectrum ranging from common cases to low-frequency, high-stakes encounters.

Following the pre-briefing, residents are directed to the simulation room and provided with the “pre-scenario” information as seen in Table 3. The nurse may add the following (passed along from the paramedic): “The patient was found on the bed, fully clothed, and very drowsy. Little is known about the patient and nothing about his medical history. His family doesn’t live in the province”.

**Table 3:** Background information and expected actions for the diabetic ketoacidosis simulation scenario AMPLE: Allergies, Medications, Past medical history, Last eaten, Events leading; BHB: beta-hydroxybutyrate; BP: blood pressure; CBC: complete blood count; DDx: differential diagnosis; EMS: emergency medical services; FHx: family history; GAEB: good air entry bilaterally; HEENT: head, eyes, ears, nose, throat; ICU: intensive care unit; IV NS: intravenous normal saline; HR: heart rate; LBC: lytes, BUN, creatinine; LFT: liver function tests; LOC: level of consciousness; MM: mucous membrane; NKDA: no known drug allergies; PMHx: past medical history; RR: respiratory rate; T: temperature; VBG: venous blood gas

Pre-scenario: You are an ER physician in a community hospital with some specialty backup. A 19-year-old male presents with weakness, nausea, vomiting, and decreased LOC. There is some dried vomit on his clothes. This is his first weekend at school.	
History:	
PMHx	Unknown
Social Hx	Newly moved to the university dormitory. Living with new roommate - doesn’t really know him at all. Has been here for two days. Alcohol last night/yesterday. The nurse will give this history if asked by a learner.
Surgical Hx	Unknown
Medication	Unknown
Allergies	NKDA
FHx	Unknown
Other	HR 130 / BP 100/60 / T35.7 / RR 24 / SpO2 98% on room air ** must ASK for glucose check.
Physical exam:	
General	Drowsy, pale.
HEENT	Non-icteric, MM dry.
Pulmonary	GAEB, tachypnea.
Cardiovascular	Tachycardia, regular, no murmur.
Abdomen	No peritonitis, mild diffuse abdominal tenderness, thin.
Breath odor	**only if resident specifically asks - fruity odour.
Investigations:	
Electrocardiography (ECG) 1 & 2	
Optional: Chest x-Ray (CXR), Point of Care Ultrasound (PoCUS)	
Case progression:	
Expected actions 1 - Primary assessment	
ABC	
Intravenous (IV) O2 monitor	
AMPLE history - Details above	
Collateral history - Limited (from roommate), EMS record; no family is present; **Parent can be contacted if they find personal information/wallet and call them.	
Physical exam - ABCDE	
Initial vitals: HR 130 / BP 100/60 / T35.7 / RR 24 / SpO2 98% on room air, ** must ASK for glucose check.	
Expected actions 2	
Order labs: CBC, LBC, LFT, amylase, lactate, coags, VBG, serum BHB, ER toxicology screen, blood cultures +/-	
Investigations: ECG 1 (tachy, peaked T waves, wide QRS), +/- PoCUS (negative), CXR (negative).	
Interventions: 2L IV NS	
If above done, GO TO 3.	
IF NOT DONE, (or less than 1-2L bolus) GO TO 4.	
Expected actions 3	
With positive actions in Step 2, repeat vitals improve somewhat:	
HR 120, BP100/60, T35.7, Sat 96% RA, RR24	
Recognize and treat hyper K- as indicated by scenario and ECG appearance (ECG1) - give Ca, fluids, salbutamol, insulin.	
Repeat ECG (if requested) - ECG 2.	
Recognize DKA.	
Initiate insulin infusion at appropriate time.	
Consider DDx, triggers for DKA & treatment of these.	
Consider HCO3 administration.	
Labs become available in latter half of case - see appendix.	
IF DONE, GO TO END 1; IF NOT DONE, GO TO 4.	
Expected actions 4:	
The patient now has vitals (deteriorated):	
HR 130 / BP 90/60 / T35.7 / RR26 / SpO2 98% RA	
Recognize worsening; initiate appropriate treatment.	
Review labs.	
If- treats k, hypotension, acidosis, DKA (insulin fluids) - go to End 1.	
If not aggressive Tx of DKA, K, hypotension, acidosis - go to End 2.	
End scenario:	
End 1 - the case can end with the successful resuscitation of the patient who stabilizes but remains in serious condition. Medicine and ICU should be consulted.	
HR 110-120, BP 100/60, T35.7, Sat 96% RA, RR20	
End 2 - Failure to resuscitate adequately. Becomes more obtunded/drowsy with deterioration of vitals and general condition.	
HR 130 / BP 80/50 / T35.7 / RR30 / SpO2 98% RA	

**Figure 1:**
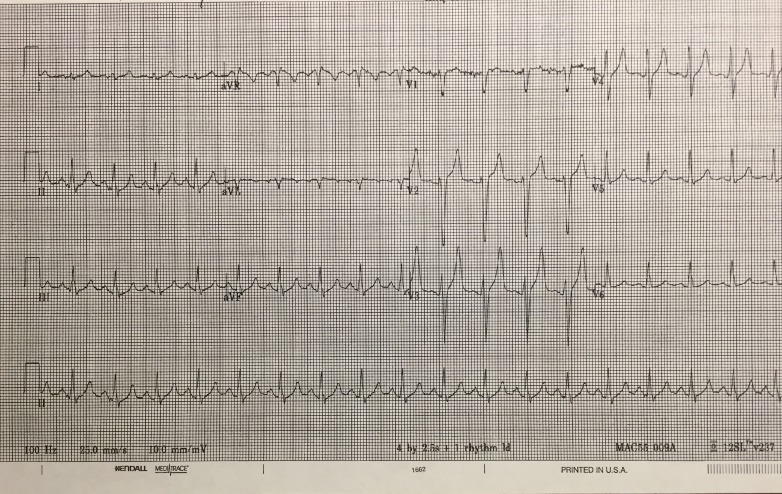
Initial electrocardiogram shows tachycardia, peaked T-waves, and QRS widening

**Figure 2:**
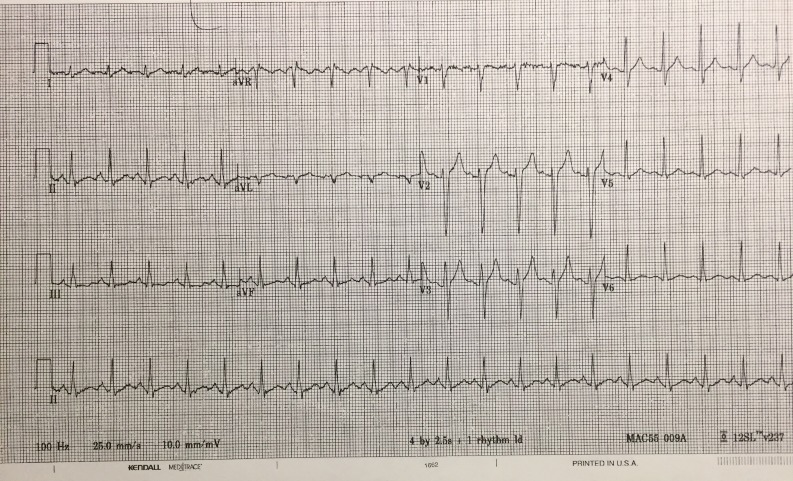
Repeat electrocardiogram

Once in the room, the students are expected to assign roles and function as a team to diagnose and treat the patient appropriately. Using available resources including the attending SP nurse, ED triage sheet, EMS record, and interaction with the patient and their family, the student will gather key information. The team lead can utilize the skills of other learners to help complete tasks. Additional help, such as specialist backup, may be requested but is often not available. Monitors and intravenous (IV) are usually not established at the beginning of the case and learners will have to request these. A full set of vitals may not be provided up front and the student must recognize this. Early interventions and investigations should be initiated (as appropriate to the case) including fluid bolus, request for electrocardiogram (ECG), blood work, and x-ray (Figures 1-2) (Table 4).

**Table 4:** Lab results for a patient with diabetic ketoacidosis BHB: beta-hydroxybutyrate; CBC: complete blood count; eGFR: estimated glomerular filtration rate; Hct: hematocrit; Hgb: hemoglobin; LBC: lytes, BUN, creatinine; MCV: mean corpuscular volume; OSM: osmolality; VBG: venous blood gas

LBC						
Test		Result	Flag			Reference
Urea		*23.9*	*H*			3 – 7 mmol/L
Sodium		*128*	*L*			135 – 145 mmol/L
Potassium		*7.6*	*H*			3.5 – 5 mmol/L
Chloride		*90*	*L*			95 – 110 mmol/L
CO2		*5*	*L*			22 – 32 mmol/L
Glucose		*65.6*	*H*			3.5 – 7.8 mmol/L
Creatinine		*214*	*H*			54 – 113 mmol/L
eGFR		32				
CBC						
Leukocytes		*27.5*	*H*		4.8 – 10.8 10^9/L	
Erythrocytes		*4.62*	*L*		4.7 – 6.1 10^9/L	
Hgb		150			140 – 180 g/L	
Hct		0.448			0.42-0.52	
MCV		96.8			80-97 FL	
Platelets		316			130 – 400 10^9/L	
Lymphocytes		*0.9*	*L*		1.5 – 4.0 10^9/L	
Monocytes		*2.3*	*H*		0.11 – 1.0 10^9/L	
Neutrophils		*24.3*	*H*		2.0 – 7.5 10^9/L	
Eosinophils		0.0			0.0 – 0.35 10^9/L	
Basophils		0.0			0.0 – 0.2 10^9/L	
VBG						
Venous pH		*7.001*		*L*	7.32 – 7.43	
Venous pCO2		*24*		*L*	38.0 – 50.0 mmHg	
Venous pO2		65			mmHg	
Venous HCO3		*5.7*		*L*	22 – 29 mmol/L	
Base Excess		-26.0			mmol/L	
Venous SpO2		*89.1*		*H*	60 – 85%	
Venous tCO2		*6.4*		*L*	23.0 – 30.0	
BHB		9.4				
OSM		389				

With an initial assessment and complete set of vitals, the learner should go through a general approach to the patient with an altered LOC and develop/verbalize an appropriate differential and treatment plan, thus meeting the requirement for Objective 1. A standard approach to primary assessment is expected. The Airway, Breathing, Circulation (ABC)-IV-O2-Monitor is generally a good start. Abnormal vitals should be addressed at this stage. The initial ECG showed tachycardia, peaked T-waves and QRS widening (Figure 1). The learner should consider the differential of this finding and verbalize concern of hyperkalemia.

As labs and investigations are available, the student should consolidate the information and proceed with a more directed treatment of DKA and its associated complications to meet Objective 2. Fluid deficits should be corrected and specific treatments to address metabolic abnormalities should be initiated. Insulin infusion should be initiated and particular attention should be paid to electrolyte abnormalities, particularly potassium and the acid-base status of the patient. Triggers for the onset of DKA should be explored and addressed. Complications including dysrhythmias should be treated appropriately. Depending on the level of case difficulty chosen for the scenario, more advanced procedures such as intubation or central line placement may be integrated through the use of hybrid setups or the high-fidelity mannequin.

Integration of SP confederates helps to satisfy Objective 3 which focuses on the CanMEDS roles of communicator, collaborator, and professional. The SP nurse may be scripted to add challenges to the case to help highlight these roles. Alternatively, an SP family member can play roles ranging from a quiet/concerned parent to a loud and disruptive individual, challenging the student to direct attention and resources to deal with both the patient and the extenuating circumstances.

Table 1 provides suggestions for a number of input modifiers that can be used for any scenario involving high fidelity or SP (including the current case) to adjust the case difficulty by dialing up or down the “signal-to-noise” ratio, depending on the needs of the learner. Table 3 outlines the general flow of the current case.

Two faculty members are generally involved in running the scenario. One directs the changes in vitals, provides prompts and acts as the “voice” of the patient when the high-fidelity mannequin is used. This individual also communicates with the SP nurse, providing direction in the case. The second faculty member is responsible for following the temporal flow of the case and taking notes of key events for the review during the debriefing session. Particular attention is given to the pre-determined objectives.

Debriefing

Debriefing is centered around the key objectives of the case and uses the advocacy-inquiry approach, focusing on the three phases as described by Rudolph, et al. [9, 14]. This portion of the session is generally longer in duration than the simulation itself and provides the opportunity for review and discussion in a small group setting. Faculty and residents who were involved in the session are present and simulated patients are also invited to give their feedback. Depending on case outcomes and progression, additional points may be selected for clarification and discussion. Particular points of interest that instructors may wish to discuss include rapid primary assessment and early intervention in the sick, undifferentiated patient. Failure to obtain a full set of vitals occasionally leads to delayed diagnosis and is more likely to happen with a junior learner. The importance of addressing abnormal vitals before a definitive diagnosis is discussed. Inability to obtain much history from the patient should prompt a further search in common areas such as online medical records, medical bracelets, and subtle contact information available from the patient’s wallet. Once the diagnosis of DKA is made, directed treatment should be established along with a definitive plan. Specific interventions and plans for disposition should be clear. Clear communication with team members, the patient, and their family is essential for the smooth progress of the case.

Post-Scenario Didactics

Post-scenario didactics are integrated into the debriefing session and focus on the key objectives of the session. The general goal is to discuss the approach and early management of the altered LOC patient. As mentioned above, the emphasis is placed on abnormal vitals and “what the patient needs at this point”, as opposed to making a final diagnosis before initiating treatment. ABC IV-O2-Monitor can help during the first encounter with the patient and a number of mnemonics have been used to help generate a differential diagnosis list. Learners are encouraged to take a practical approach by collecting full vitals and pertinent facts to help guide further actions. Overall, the treatment of DKA is reviewed in some detail. Additional focus is placed on the sicker patient with an altered LOC, acidosis, and profound electrolyte abnormalities, often with key ECG findings. The treatment of patients of different age groups is compared and reviewed with particular attention to insulin and fluid administration.

The non-medical expert CanMEDS roles are discussed, emphasizing them as a key part of effective team function. In general, this discussion reviews clear, concise, closed-loop communication and integrates references to difficulties or challenges that may have arisen during the case. As a general rule, a more experienced learner will demonstrate greater proficiency in these areas; however, it is important to include these challenges as it will help identify areas of improvement that may be hard to pick up during day-to-day clinical rotations and encounters.

Key summary information is provided to learners after the session is completed. This may include relevant articles, checklists, online resources, or important sections in key textbooks [2-3, 15]. Without timely follow-up on the learning objectives and outcomes of the session, learners may lose out on the opportunity to consolidate and retain what they have learned.

Recognizing that important issues may arise during the case, there is flexibility in our approach and we adjust the plan to accommodate discussion around these topics. 

### Product

Expected outcomes focus on the CanMEDS roles and integrate both medical expert and non-medical expert roles [16]. Points on crisis resource management (CRM) are often integrated as a learning objective. Faculty, staff, or SP confederates can add additional challenges to the scenario, tying in the communicator, collaborator, and professional roles which the students should be familiar with.

Medical Expert: Approach to the patient with an undifferentiated altered LOC.

Medical Expert: Approach to diagnosis and treatment of DKA.

Communicator: Emphasis is placed on the appropriate use of closed-loop communication to ensure smooth team functioning in the case.

Collaborator: The student should use available resources to diagnose and treat the patient while dealing with any challenges that may arise during the case.

Professional: Respectful and effective communication is essential for efficient team performance and for addressing interpersonal difficulties introduced through the inclusion of confederates.

## Discussion

With the current focus on competency-based performance and linked milestones versus the traditional time-based approach to completion of training, simulation enables instructors to challenge learners with relevant cases and content in a timely manner, to ensure they gain proficiency. Waiting for hands-on exposure to core cases/topics in the clinical ED setting can prove inefficient and often falls short of what is needed. Patient safety and improved outcomes are the driving forces for this change [17].

This simulation scenario was developed to help learners appropriately recognize and treat a patient with DKA. A number of case modifiers are included, allowing the case difficulty to be altered depending upon the level of learners. In this case, the initially non-specific presentation of the patient with an altered level of consciousness requires the learner to step back and cast a wide diagnostic net. Once a definitive diagnosis is established, more directed treatment is necessary. Core emergency medicine references are highlighted for reviewing key information on these topics [2-3]. Learners must be aware of potential biases that can be encountered which can lead them off course from reaching an accurate diagnosis. In this case, the patient’s recent alcohol consumption was a confounding variable and was not directly responsible for the altered LOC but did lead to nausea, vomiting, and dehydration. The integration of scripted SP is an effective way to add challenges to a case and to evaluate non-medical expert CanMEDS roles which are essential for competent performance as a medical professional [16].

In more advanced versions of the scenario, procedural skills are also integrated. Endotracheal intubation and central line placement are important skills for emergency medicine physicians, and the simulation provides a safe, low-risk environment for learners to practice these skills. The inclusion of these skills usually requires a hybrid case set-up using task trainers; the didactic review will address the relevant background information and the discussion of hands-on performance [15]. Adaptation of the case to a low-fidelity setup may help to address the issues of cost and time effectiveness of a high-fidelity simulation-based training that are often prohibitive.

## Conclusions

This technical report describes the design and implementation of a simulation scenario on DKA for emergency medicine trainees. A number of key modifiers are described that allow for the adjustment of case difficulty and enable assessment of a number of CanMEDS roles. Relevant points about CRM and effective communication are also covered. Simulation provides a safe, controlled environment where learners can implement/practice their knowledge and skills to effectively train on low-frequency, high-stakes encounters and procedures as well as core topics.
